# Massive Occurrence of the Harmful Benthic Dinoflagellate *Ostreopsis* cf. *ovata* in the Eastern Adriatic Sea

**DOI:** 10.3390/toxins11050300

**Published:** 2019-05-25

**Authors:** Živana Ninčević Gladan, Jasna Arapov, Silvia Casabianca, Antonella Penna, Giorgio Honsell, Valentina Brovedani, Marco Pelin, Luciana Tartaglione, Silvio Sosa, Carmela Dell’Aversano, Aurelia Tubaro, Ante Žuljević, Branka Grbec, Matea Čavar, Mia Bužančić, Ana Bakrač, Sanda Skejić

**Affiliations:** 1 Institute of Oceanography and Fisheries, Šetalište Ivana Meštrovića 63, 21000 Split, Croatia; nincevic@izor.hr (Ž.N.G.); zuljevic@izor.hr (A.Ž.); grbec@izor.hr (B.G.); buzancic@izor.hr (M.B.); jelacic@izor.hr (A.B.); sanda@izor.hr (S.S.); 2 Department of Biomolecular Sciences, University of Urbino, Viale Trieste 296, 61121 Pesaro, Italy; silvia.casabianca@uniurb.it (S.C.); antonella.penna@uniurb.it (A.P.); 3 Consorzio Nazionale Interuniversitario per le Scienze del Mare (CoNISMa), Piazzale Flaminio 9, 00196 Roma, Italy; luciana.tartaglione@unina.it (L.T.); dellaver@unina.it (C.D.); 4 Department of Agricultural, Food, Environmental and Animal Sciences, University of Udine, Via delle Scienze 91/93, 33100 Udine, Italy; giorgio.honsell@uniud.it; 5 Department of Life Sciences, University of Trieste, Via A. Valerio 6, 34127 Trieste, Italy; valentina.brovedani@phd.units.it (V.B.); mpelin@units.it (M.P.); ssosa@units.it (S.S.); tubaro@units.it (A.T.); 6 Department of Pharmacy, University of Napoli, Federico II, Via D. Montesano 49, 80131 Napoli, Italy; 7 Department of Marine Studies, University of Split, Ruđera Boškovića 37, 21000 Split, Croatia; cavarmatea1@gmail.com

**Keywords:** Adriatic Sea, *Ostreopsis ovata*, *Coolia monotis*, ovatoxin, palytoxin

## Abstract

In September 2015, a massive occurrence of the *Ostreopsis* species was recorded in central Adriatic Kaštela Bay. In order to taxonomically identify the *Ostreopsis* species responsible for this event and determine their toxin profile, cells collected in seawater and from benthic macroalgae were analyzed. Conservative taxonomic methods (light microscopy and SEM) and molecular methods (PCR-based assay) allowed the identification of the species *Ostreopsis* cf. *ovata* associated with *Coolia monotis*. The abundance of *O.* cf. *ovata* reached 2.9 × 10^4^ cells L^−1^ in seawater, while on macroalgae, it was estimated to be up to 2.67 × 10^6^ cells g^−1^ of macroalgae fresh weight and 14.4 × 10^6^ cells g^−1^ of macroalgae dry weight. An indirect sandwich immunoenzymatic assay (ELISA) and liquid chromatography–high-resolution mass spectrometry (LC-HRMS) were used to determine the toxin profile. The ELISA assay revealed the presence of 5.6 pg palytoxin (PLTX) equivalents per *O.* cf. *ovata* cell. LC-HRMS was used for further characterization of the toxin profile, which showed that there were 6.3 pg of the sum of ovatoxins (OVTXs) and isobaric PLTX per *O.* cf. *ovata* cell, with a prevalence of OVTXs (6.2 pg cell^−1^), while the isobaric PLTX concentration was very low (0.1 pg cell^−1^). Among OVTXs, the highest concentration was recorded for OVTX-a (3.6 pg cell^−1^), followed by OVTX-b (1.3 pg cell^−1^), OVTX-d (1.1 pg cell^−1^), and OVTX-c (0.2 pg cell^−1^).

## 1. Introduction

In the last two decades, massive occurrences of the benthic dinoflagellate *Ostreopsis* species in different parts of the Mediterranean Sea have been well documented (Table 1). Three *Ostreopsis* species have been recorded so far in various Mediterranean regions: *Ostreopsis* cf. *ovata*, *Ostreopsis* cf. *siamensis* [1,2,3,4,5,6,7], and *Ostreopsis fattorussoi* [8].

**Figure 1:**
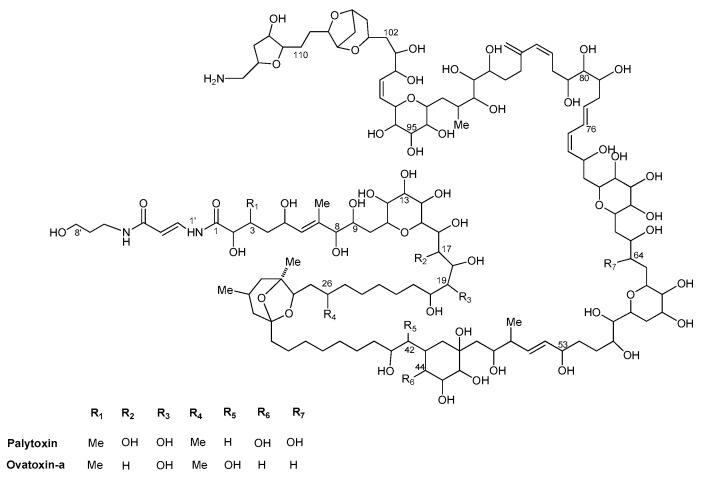
Chemical structures of palytoxin and ovatoxin-a, the latter being found in Mediterranean *Ostreopsis* cf. *ovata* strains.

*Ostreopsis* blooms in the Mediterranean Sea are commonly accompanied by respiratory problems and skin irritation in humans exposed to marine aerosol containing algal toxins and/or cells debris and seawater [9,10,11,12] due to ability of some *Ostreopsis* species to produce toxins. Most of these toxins belong to the palytoxin (PLTX) group. PLTX and its analogs may affect human health by ingestion of contaminated seafood, skin contact with seawater, and inhalation of marine aerosols containing *Ostreopsis* cell debris and/or their toxins [12,13,14]. So far, human poisonings ascribed to ingestion of PLTX-contaminated seafood have been recorded in the Pacific and Indian Oceans following consumption of fish [13,15,16,17,18,19] and crabs [20,21], while in the Mediterranean Sea, no cases have been reported so far. Chemical studies of the Mediterranean strains of *O.* cf. *ovata* showed the presence of small quantities of an isobaric PLTX [22,23] and larger amounts of structural PLTX congeners called ovatoxins (OVTXs), with a high prevalence of OVTX-a (Figure 1) [24,25,26,27]. According to Funari et al. [28], the lack of toxicity in humans via food chain transfer during *O.* cf. *ovata* blooms in the Mediterranean area could be explained by the lower oral toxicity of OVTXs in comparison with that of PLTXs. This hypothesis has been supported by the in vitro cytotoxicity characterization of OVTX-a in relation to the reference compound PLTX, which revealed less toxic effect compared with PLTX, displaying lower cytotoxicity as well as lower hemolytic activity on human erythrocytes [29].

According to literature (Table 1) the most abundant and widely distributed *Ostreopsis* species in the Adriatic Sea is the Atlantic/Mediterranean ribotype *O*. cf. *ovata* [7]. The first identification of *O.* cf. *ovata* in Croatian waters was from the central Adriatic Kaštela Bay in 1984 [30]. Thereafter, this species was not reported in Kaštela Bay, but it is possible that it remained undetected due to the absence of visible blooms and the lack of noted negative impacts on human health. *Ostreopsis* cf. *ovata* has been reported along the Italian coasts since the late 1990s and, with a few exceptions, almost all Italian regions are seasonally affected by *O*. cf. *ovata* blooms [28]. Cases of respiratory problems and skin irritations in humans associated with massive blooms of *O.* cf. *ovata* in Croatian waters were reported for the first time in the northern Adriatic Sea in 2010 [31]. In 2015, complaints of similar signs and symptoms came from the beaches along the Kaštela Bay coasts, indicating the development of another *O*. cf. *ovata* bloom, this time in the central part of the Adriatic Sea.

Based on this event, the aim of this study is: (1) Taxonomical identification of the *Ostreopsis* species that cause massive blooms and affect human health; (2) determination of the toxin profile of these species; (3) reporting a new site where blooms occur to improve global mapping of the genus *Ostreopsis*; and (4) raising awareness about the necessity of introducing targeted monitoring of *Ostreopsis* species by reporting its occurrence.

## 2. Results

### 2.1. Microscopy Analyses

**Figure 2:**
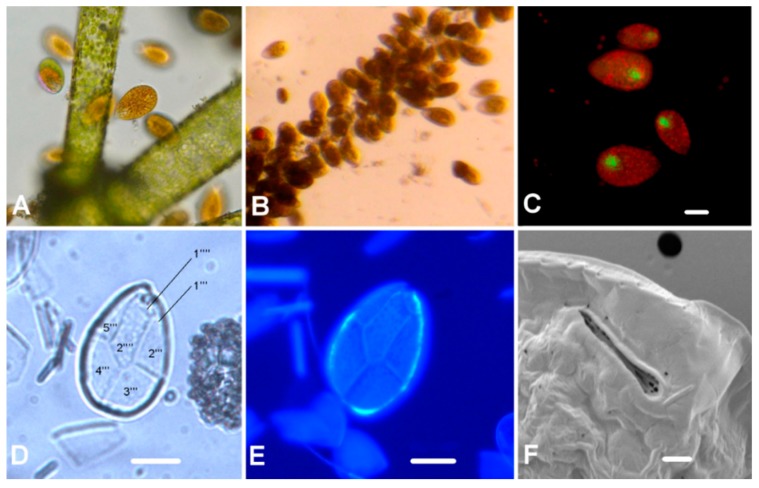
*Ostreopsis* cf. *ovata*: (**A**,**B**) Live samples under light microscope, (**C**) nucleus dyed with SYBR Green 1, (**D**) antapical view with plate tabulation, (**E**) epifluorescence observation of cell after Calcofluor White staining, and (**F**) apical pore (Po) detail under SEM. Scale bars for (**A**–**E**) are 20 µm, and scale bar for (**F**) is 2 µm.

Microscopic analyses of field samples showed an intensive bloom of *Ostreopsis* species (Figure 2A–C). *Ostreopsis* cf. *ovata* was identified on the basis of its specific cellular shape (like a pumpkin seed with an expanded oval dorsal side and a narrowed ventral part), morphological characteristics, and the ratio of dorsoventral (DV) and anteroposterior (AP) diameter (Table 2). The ventral portion is characterized by a protrusion which is usually less pigmented due to mucus material.

**Figure 3:**
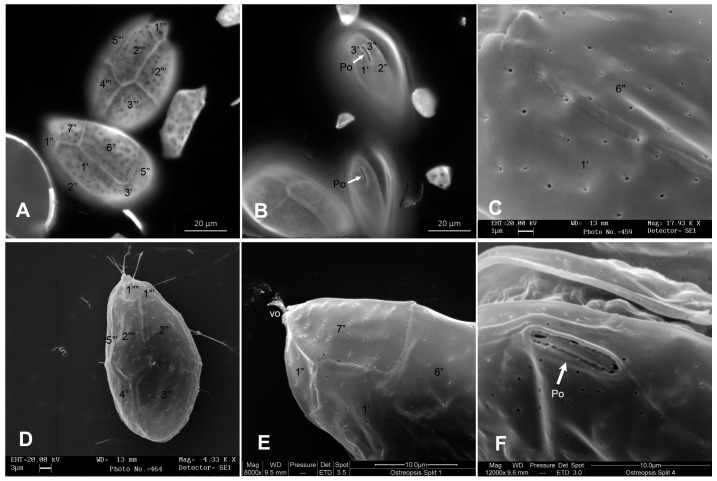
*Ostreopsis* cf. *ovata:* (**A**) Hypotheca and epitheca observed by epifluorescence microscopy after staining with Calcofluor White M2R, (**B**) epithecae showing pore plate Po (arrow) observed by epifluorescence microscopy after staining with Calcofluor White M2R, and (**C**) thecal plates observed by scanning electron microscopy. The surface appears smooth and perforated by many small pores; scale bar is 1 µm. (**D**) Hypotheca observed by scanning electron microscopy; scale bar is 3 µm. (**E**) Epitheca ventral view showing the ventral opening (vo): Filamentous material appears to be discharged through it, confirming its role in mucilage release; scale bar is 10 µm. (**F**) Epitheca view showing the apical pore plate (Po); scale bar is 10 µm.

Epifluorescence and SEM microscopy showed a plate pattern Po 3’7’’5’’’2’’’’ (Figure 2D,E and Figure 3A,B,D,E) that fit well with the original description. The average DV/AP ratio was 2.17 (±0.20), ranging from 1.57 to 2.54. The apical pore plate (Po) average length was 8.59 μm (±0.70), ranging from 6.72 to 10.05 μm (*n* = 65) (Figure 2F and Figure 3F). Thecal plates were smooth with small pores (0.16–0.24 μm) scattered over their surface (Figure 3C). The nucleus, with an average width of 8.70 µm (±1.31) (*n* = 56), occupied a dorsal position in the cell (Figure 2C).

### 2.2. Molecular Analyses

**Figure 4:**
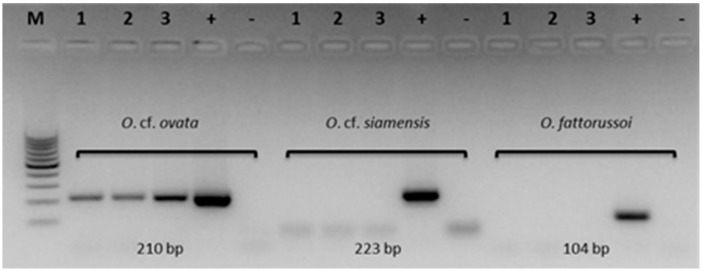
Species-specific PCR amplification of *O.* cf. *ovata, O.* cf. *siamensis*, and *O. fattorussoi* on filter samples (1, 2, and 3,) from Kaštela Bay using species-specific primers designed on ITS-5.8S rDNA; positive control of clonal culture *O*. cf. *ovata* CBA 3041, *O*. cf. *siamensis* CBA CNR-T5, and *O*. *fattorussoi* CBA L1000 (+); negative control with sterile water (−). M, 100 bp DNA Ladder molecular size marker.

The molecular PCR amplifications were carried out on field samples to detect the presence of the species *O.* cf. *ovata* together with *O.* cf. *siamensis* and *O. fattorussoi*. All these *Ostreopsis* species are present along the coast of the Mediterranean Sea [7,80,81]. These environmental samples contained mixed microphytobenthic assemblages including target taxa. Only the PCR amplified products of expected sizes of *O*. cf. *ovata* (210 bp) were detectable in the environmental samples containing *Ostreopsis* spp. cells. A PCR-based assay identified only *O.* cf. *ovata* in the environmental samples, for which species-specific identification of *Ostreopsis* cells proved quite difficult using LM or needed taxonomical identification confirmation with negative PCR amplification for *O.* cf. *siamensis* and *O. fattorussoi* (Figure 4).

### 2.3. Ostreopsis cf. ovata Abundance and Phytoplankton Community Composition

The abundance of *O.* cf. *ovata* in seawater in September ranged from 1.5 × 10^4^ to 2.9 × 10^4^ cells L^−1^. Epiphytic cells recorded on macroalgae ranged from 2.25 × 10^6^ to 2.67 × 10^6^ cells g^−1^ of fresh weight of macroalgae and 11.4 × 10^6^ to 14.4 × 10^6^ cells g^−1^ of dry weight of macroalgae. The analysis of the benthic macroalgal assemblage showed the prevalence of the red macroalga *Spyridia filamentosa* (Wulfen) Harvey (1833) in the sampling area. The maximum abundance of *O*. cf. *ovata* was recorded in September during calm weather and with a surface seawater temperature of 23.4 °C. At the beginning of October, with the surface seawater temperature decreasing to 20.8 °C and SE winds of 2 Bf, the abundance of *O*. cf. *ovata* decreased by an order of magnitude, with abundances in seawater from 1.28 × 10^3^ to 1.92 × 10^3^ cells L^−1^. Abundances of epiphytic cells of *O*. cf. *ovata* on macroalgae also decreased by an order of magnitude and ranged from 1.66 × 10^5^ to 4.29 × 10^5^ cells g^−1^ of fresh weight of macroalgae and 6.99 × 10^5^ to 2.66 × 10^6^ cells g^−1^ of dry weight of macroalgae.

In September, the phytoplankton community in the seawater was dominated by the diatoms *Pseudo-nitzschia* spp.; *Chaetoceros* sp.; *Guinardia delicatula* (Cleve) Hasle, 1997; *G. striata* (Stolterfoth) Hasle, 1996; *Leptocilyndrus danicus* Cleve, 1889; *Navicula* sp.; and *Pleurosigma* sp. (Table 3). The contribution of *O.* cf. *ovata* cells in the water column (2.9 × 10^4^ cells L^−1^) in September represented up to 10% of the total phytoplankton community, decreasing to less than 1% in October with abundances up to 1.9 × 10^3^ cells L^−1^. October was also characterized by a strong prevalence of diatoms in the phytoplankton community.

**Figure 5:**
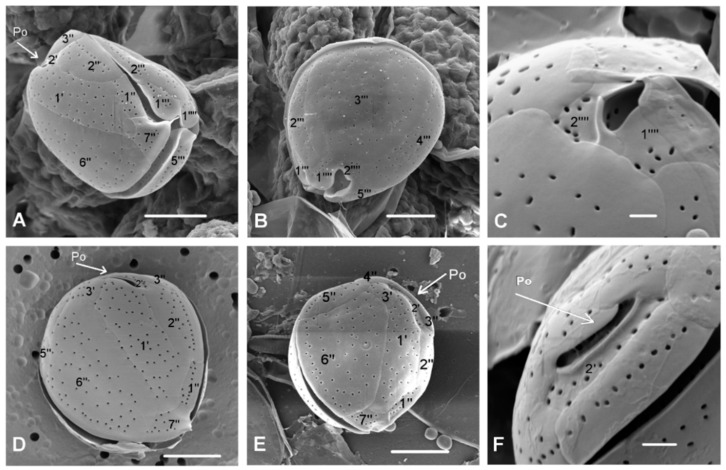
SEM micrographs of *Coolia monotis* with plate tabulation: (**A**,**D**,**E**) Apical view, (**B**,**C**) antapical view, and (**F**) apical pore (Po) detail. Scale bars for (**A**,**B**,**D**,**E**) are 10 µm; scale bars for (**C**,**F**) are 2 µm.

*Ostreopsis* cf. *ovata* was accompanied by the epiphytic dinoflagellate *Coolia monotis*. Taxonomical identification of *C. monotis* was based on size and morphological features obtained by SEM (Figure 5). Tabulation was determined according to Balech [82]. Plate 7’’ is characterized by the ratio of width and length of approximately 1. Plate 1’ is placed left of the center. While the abundances of *C. monotis* in seawater were lower than those of *Ostreopsis* cells, they were of the same order of magnitude throughout the sampling period. In September, abundances in seawater ranged from 3.20 × 10^2^ to 1.12 × 10^3^ cells L^−1^. Epiphytic cells recorded on macroalgae ranged from unrecorded to 2.67 × 10^5^ cells g^−1^ of fresh weight of macroalgae and unrecorded to 1.15 × 10^6^ cells g^−1^ of dry weight of macroalgae. In October, abundances of *C. monotis* in seawater ranged from 4.80 × 10^2^ to 1.6 × 10^3^ cells L^−1^. Epiphytic cells recorded on macroalgae ranged from 2.68 × 10^4^ to 1.35 × 10^5^ cells g^−1^ of fresh weight of macroalgae and 1.12 × 10^5^ to 8.35 × 10^5^ cells g^−1^ of dry weight of macroalgae.

### 2.4. Concentration and Characterization of Ostreopsis Toxins

**Figure 6:**
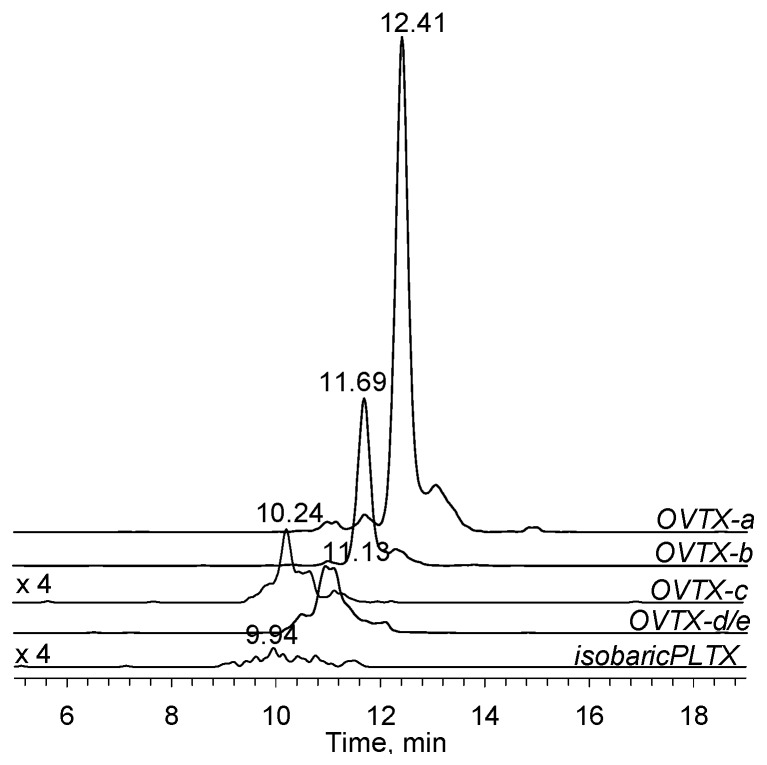
Extracted ion chromatograms (XICs) of all the palytoxin (PLTX) congeners (ovatoxin (OVTX)-a to -e and isobaric PLTX) identified in the analyzed *Ostreopsis* cf. *ovata* extract obtained by selecting the most abundant peak of [M+H+Ca]^3+^ ion (mass tolerance = 5 ppm) of each toxin, namely: OVTX-a *m*/*z* 896.1572, OVTX-b *m*/*z* 910.8318, OVTX-c *m*/*z* 916.1628, OVTX-d/e *m*/*z* 901.4884, and isobaric PLTX *m*/*z* 906.8167.

**Figure 7:**
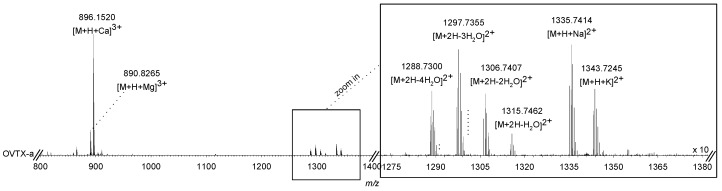
Liquid chromatography–high-resolution mass spectrometry (LC-HRMS) spectrum of ovatoxin-a, the main component of the *O.* cf. *ovata* profile. The spectrum is dominated by the triply charged calcium adduct ion and contains a number of doubly charged ions.

An indirect sandwich immunoenzymatic assay (ELISA) for palytoxin detection carried out on field microalgal samples showed 5.6 pg PLTX equivalents per *Ostreopsis* cell. A parallel investigation of the detailed toxin profile of *O.* cf. *ovata* was carried out by LC-HRMS and quantitative results were compared. Extracted ion chromatograms (XICs) for all the known PLTX congeners revealed the presence of OVTX-a–e and isobaric palytoxin (Figure 6), the identity of which was ascertained by: (i) Comparison of the retention times of individual compounds with those of ovatoxins contained in a reference sample available at the University of Naples Federico II; (ii) the diagnostic ion profile of ovatoxins and palytoxin analogs contained in full HRMS spectra (mass range *m*/*z* 800–1400) of each molecule, which represents a fingerprint for this class of molecules (Figure 7); and (iii) elemental formula assigned to the monoisotopic ion peak of each ion (mass tolerance < 3 ppm) and isotopic pattern. The total toxin content measured by LC-HRMS was 6.3 pg cell^−1^ (Table 4) with OVTX-a being the major component, accounting for 57.1% of the total toxin content, followed by OVTX-b (20.6%), OVTX-d/e (17.5%), OVTX-c (3.2%), and isobaric PLTX (1.6%).

## 3. Discussion

For the first time, the benthic dinoflagellates *O.* cf. *ovata* and *C. monotis* from Kaštela Bay were morphologically characterized. Both species were identified on the basis of morphological features, including thecal plate pattern, shape, and size. The thecal plate tabulation of *O.* cf. *ovata* cells described in this study (Po 3’7’’5’’’2’’’’) fit well with the original description by Fukuyo [83]. The designation of the thecal plates of *O.* cf. *ovata* have changed and been reinterpreted over time. Besada et al. [84] redetermined the first precingular plate determined by Fukuyo [83] as the first apical plate and completed the formula with sulcal and cingular plates (Po 4’6’’6C8S5’’’2’’’’). This new designation that considered the homology of the plates more than the relationship with the apical pore was supported by Fraga et al. [85] and Escalara et al. [86]. In this study, we adopted the original tabulation by Fukuyo [83], which is in accordance with Kofoidean plate nomenclature and accepted by most authors with slight modifications [6,87,88,89]. The identification of *O.* cf. *ovata* was further confirmed by the DV/AP ratio, which is for *O. siamensis* either higher than 4 according to Penna et al. [6] or about 3 according to Aligizaki and Nikolaidis [43], as opposed to the congeneric species *O.* cf. *ovata*, which is characterized by a DV/AP ratio lower than 2. In Kaštela Bay, *O.* cf. *ovata* was accompanied by *C. monotis* as it has been observed in other Mediterranean areas [1,4,34,43,53,67,90,91] where *O.* cf. *ovata* appeared in association with other benthic dinoflagellates, such as *C. monotis* and *Prorocentrum lima.*

In addition, the identification of *O.* cf. *ovata* was confirmed by molecular PCR amplification using species-specific primers. In fact, due to the morphological plasticity and variability of *Ostreopsis* cells with consequent difficulty of species-specific identification, a PCR-based assay was applied to field samples in order to accurately identify the *Ostreopsis* species, which confirmed the microscopy analysis [69,92,93]. The molecular PCR assay is widely and successfully used because it is accurate, rapid, and reliable when applied to environmental samples [3,94]. It was found that only *O.* cf. *ovata* was present in the analyzed samples.

In order to determine the toxin profile of the *Ostreopsis* species found in Kaštela Bay, we used an indirect sandwich immunoenzymatic assay (ELISA) and LC-HRMS. While ELISA allowed us to measure the total toxin content (5.6 pg PLTXeq cell^-1^), LC-HRMS analyses provided the individual and total toxin contents. As a result, 6.3 pg of the sum of OVTXs and isobaric PLTX per *Ostreopsis* cell was measured, showing a prevalence of OVTX-a (3.6 pg cell^−1^). A comparison between the measurements made by the two approaches (LC-HRMS and ELISA) points to a toxin content of the same order of magnitude. However, due to a lack of replicates, no actual correlation can be extrapolated from the data. These toxin concentrations are similar to those recorded in *Ostreopsis* cells from the Ligurian Sea [24] but significantly lower than those recorded in the algal cells from the Conero Riviera (NW Adriatic), Catalan Sea, and NE Adriatic Sea [31,67,68] or those obtained from cultured *Ostreopsis* cells [95]. The absence of human poisoning associated with seafood consumption during *O*. cf. *ovata* blooms in the Mediterranean area could be tentatively related to the lower oral toxicity of ovatoxins (mainly OVTX-a) with respect to that of PLTX, as suggested by in vitro studies showing that OVTX-a cytotoxicity is about 100-fold lower than that of PLTX and also has lower hemolytic potency [29]. Nevertheless, the toxin content in *O*. cf. *ovata* cells recorded in Kaštela Bay could be related to the health problems recorded in humans exposed to marine aerosol and/or directly to seawater concomitantly with *Ostreopsis* bloom.

In the last two decades, *Ostreopsis* blooms have become common in the Mediterranean Sea, regularly occurring during the summer–autumn period (Table 1). According to the available literature, the highest abundances of *Ostreopsis* species in the Mediterranean Sea were recorded in the Ligurian Sea, along the Marche and Apulia coasts in the Adriatic Sea, the Balearic Sea, and the Catalan Sea. The highest abundances of *Ostreopsis* cells on macroalgae were reported in 2008 and 2009, while the highest abundances in seawater were reported in 2006, 2010, and 2016. It is interesting to note that all the reported maximal abundances of *Ostreopsis* species listed in Table 1, occurred during the negative phase of the North Atlantic Oscillation (NAO) index. The exception was in 2016, when blooms occurred during the positive phase of the NAO index, but this was preceded by a strong negative phase. A negative phase of the NAO index is characterized by a reduced pressure gradient, resulting in fewer and weaker winter storms that bring moist air into the Mediterranean. The analysis of precipitation data along the Croatian coast has shown a significant negative correlation with the NAO index [96].

In comparison with previously reported *Ostreopsis* occurrence in the Mediterranean Sea (Table 1) the abundance of epiphytic cells of *O.* cf. *ovata* recorded in this study was one of the highest recorded abundances and was accompanied by citizen complaints. At the same time, in the summer of 2015, a massive occurrence of *Ostreopsis* species in the northern Adriatic near Rovinj, Croatia was recorded by a scientist from the Ruđer Bošković. Many complaints from citizens on a Facebook page that was opened regarding that event were received. Several years ago, there was a mass appearance of *Ostreopsis* species in the same area in the vicinity of Rovinj [31]. These findings point to the importance of introducing beach monitoring regarding the presence of *Ostreopsis* bloom along the Croatian coast, as is already done along the Italian coast [79].

**Figure 8:**
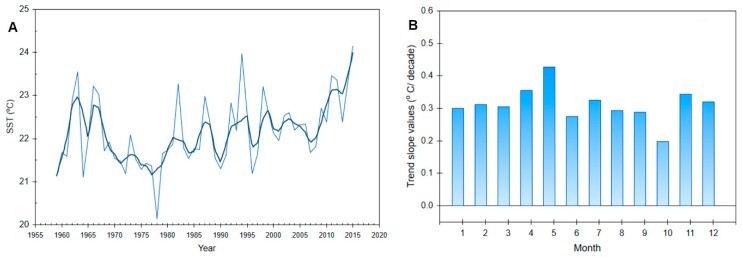
(**A**) Time series of the SST values for the summer period (JAS) and (**B**) associated linear trends at the Split coastal station according to Grbec et al. [99].

Since *Ostreopsis* sp. bloom events are commonly associated with summer periods, some authors have proposed global warming as being the determining influence on *Ostreopsis* events [97,98]. The reported bloom of *O.* cf. *ovata* in Kaštela Bay in 2015 was associated with a trend of increasing sea surface temperatures in the bay. A linear trend analysis of sea surface temperature in the area of the eastern middle Adriatic shows the existence of an upward summer sea surface temperature trend (July–September) (Figure 8). In the last few decades (1979–2015), a positive trend has been observed in the entire Eastern Adriatic Sea [99], with several records of extreme sea surface temperatures in the warming season as a result of heat waves passing over Europe. Those heat waves hit Europe, North Africa, and the Middle East in the late spring and summer, where many new temperature records were measured. The heat continued in September, spreading across Eastern Europe. Modeling experiments suggest that anthropogenic forcing was a major factor in setting the conditions for the development of the 2015 heat wave [100]. According to the Croatian National Meteorological and Hydrological Service (DHMZ), the summer of 2015 in the middle Adriatic was generally dry, except for a rainy August, compared with the climatological average (http://meteo.hr/index_en.php).

In contrast, some studies have shown that the growth of this species is not exclusively related to temperature [2]. These results are supported by the fact that in various parts of the Mediterranean, blooms of *Ostreopsis* appeared in different seasons contrary to the expectations regarding to the summer temperature of sea water [2,4]. Namely, according to the previous studies and the results of this research, the bloom in the Adriatic occurred in September, while in the Ligurian and Tyrrhenian Seas highest cell abundances are reported to occur earlier, in midsummer (July and August) [22,48,62] although summer temperatures are higher in the Adriatic Sea [2]. Based on these findings Mangalajo et al. [2] hypothesized that threshold temperature is required for *Ostreopsis* proliferation and maximal abundance is site specific related beside the temperature with others environmental factors as nutrients, substrate characteristic including macroalgal communities, biotic interactions as well as waves and currents. Hydrodynamics is an important factor involved in the ending of *Ostreopsis* bloom as demonstrated by earlier reports and results of this study. The observed intense bloom of *O.* cf. *ovata* in Kaštela Bay occurred during calm weather in September and decreased by an order of magnitude over a 12-day period, with lower temperatures and windy weather in October, confirming the importance of specific hydrodynamic conditions for the dynamics of *Ostreopsis* blooms previously reported by Accoroni and Totti [81]. The significant effect of hydrodynamics for *Ostreopsis* cells in seawater was confirmed by an investigation in the Ligurian Sea, while benthic stocks seem much more resistant to wave motion [101]. The benthic stock in this study also decreased by an order of magnitude but still remained high.

## 4. Conclusions

*Ostreopsis* species are generally occurred in tropical waters, but its occurrence spread world-wide and its massive occurrence is well documented in the Mediterranean Sea (Table 1). Kaštela Bay is reported as a new site where *O*. cf. *ovata* blooms occurred causing the brown floating aggregate appeared in shallow parts. Since a massive occurrence of this species was recorded in the northern Adriatic Sea (near Rovinj) in the same year that this bloom occurred, as well as a few years ago, a monitoring program of toxic *Ostreopsis* species along the eastern Adriatic coast should be introduced to prevent health problems. OVTX-a was found to be the dominant toxin in the toxin profile, accounting for 57.1% of the total toxin content followed by OVTX-b (20.6%), OVTX-d/e (17.5%), OVTX-c (3.2%), and isobaric PLTX (1.6%), which was in good agreement with the toxin profile identified in the frame of a previous study on a number of different Mediterranean *O.* cf. *ovata* strains [102].

## 5. Materials and Methods

### 5.1. Sampling

**Figure 9:**
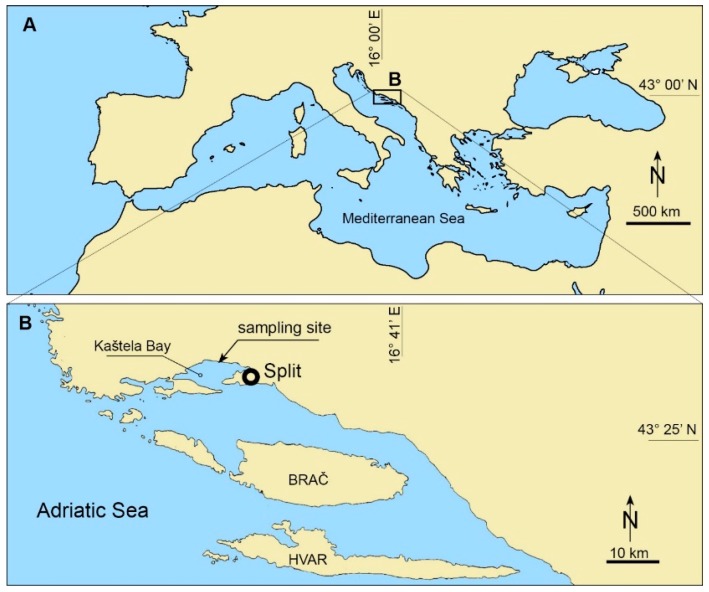
Central Adriatic shoreline with the location of the recorded *Ostreopsis* bloom.

Based on complaints of citizens about adverse effects associated with the bloom of *Ostreopsis*, sampling of phytoplankton and the macroalgal community was performed in September and October 2015 near the beach in Kaštela Bay (Figure 9). Seawater samples were taken by a Niskin sampler to determine the abundance of *Ostreopsis* spp. cells. Substrate macroalgae at a 1-m depth were scraped from stones using a rectangular frame (20 × 20 cm) and shaken in 6 L of seawater. Seawater samples and substrate macroalgae were taken in triplicates with about a 3-m distance, making a total of 12 samples. Two subsamples of each final shake were fixed for the taxonomical identification of *Ostreopsis*, using both light and electron microscopes. The rest of the final shake was filtered by gravity on a 0.45-μm filter (Millipore membrane filters) to separate algal cells from the seawater. Pellets on the filter were frozen at −20 °C for subsequent toxin analyses and taxonomic identification by molecular analyses.

### 5.2. Microscopy Determinations

Phytoplankton community composition and abundance were analyzed according to the Utermöhl method [103]. Taxonomic identification of *Ostreopsis* species was performed using epifluorescence microscopy after Calcofluor treatment and SEM. For epifluorescence microscopy, cells were fixed with 2% EM-grade glutaraldehyde dissolved in filtered seawater, stained with Calcofluor White M2R (Sigma-Aldrich, St. Louis, MO, USA) and SYBR Green (Lonza, Rockland, ME, USA)), and observed at 400× magnification using the epifluorescence microscope Zeiss AxioObserver Z1 (Carl Zeiss AG, Oberkochen, Germany) with Zeiss Filter Set 34 (excitation: 379–401 nm, emission: 435–485 nm, and beam splitter: 420 nm) and image acquisition by a Zeiss AxioCam MR M3 camera and epifluorescence microscope Leica DMI4000 B (Leica Microsystems; Wetzlar, Germany). For SEM observations, samples were preserved with 2% EM-grade glutaraldehyde, which was dissolved in filtered seawater. Subsequently, the samples were washed in 1:1 seawater/distilled water and then in distilled water. After that, samples were dehydrated in a gradual series of ethanol solutions and then critical-point dried with liquid carbon dioxide. Finally, samples were sputter-coated with gold and observed with LEICA STEREOSCAN 430i (Leica Microsystems; Wetzlar, Germany), FEI Quanta 200 (FEI, Thermo Fisher Scientific, Hillisbo, OR, USA), and MIRA 3 (Tescan, Brno, Czech Republic) scanning electron microscopes.

### 5.3. Molecular Analyses

Filter samples containing *Ostreopsis* cells were rinsed with sterile filtered seawater, the recovered volume (4 mL) was centrifuged at 4000× *g* for 10 min, and the supernatant was discharged. A second rinse with 1 mL of sterile filtered seawater was performed and the suspension was centrifuged at 1000× *g* for 10 min. Total genomic DNA was extracted from the obtained cell pellets using the DNeasy Plant Kit, and species-specific PCR assays for *O.* cf. *ovata, O.* cf. *siamensis*, and *O. fattorussoi* were carried out by amplifying 1 ng of genomic DNA according the protocols described by Battocchi et al. [3] and Vassalli et al. [104]. Expected amplicon size were 210, 223, and 104 base pair (bp) for *O.* cf. *ovata, O.* cf. *siamensis*, and *O. fattorussoi*, respectively. The PCR products were resolved on a 1.8% (*w*/*v*) agarose gel, 1× TAE buffer gel and were visualized by GelRed staining under UV light.

### 5.4. Chemical and Immunoenzymatic Analyses

#### 5.4.1. Extraction

A cell pellet was added to 3 mL of methanol/water (1:1, *v*/*v*) and extraction was performed by pulse sonication for 10 min in an ice bath. Centrifugation (6500 rpm for 1 min) was carried out to separate the supernatant from the residue. The extraction procedure was repeated twice on the pellet with 2 mL of methanol/water, combining the extracts to a final volume of 7 mL. The extract was analyzed by an indirect sandwich immunoenzymatic assay and liquid chromatography–high-resolution mass spectrometry.

#### 5.4.2. Indirect Sandwich Immunoenzymatic Assay (ELISA)

The microalgal extract was analyzed by an indirect sandwich ELISA, as described by Boscolo et al. [105]. Briefly, ELISA multiwell strips were coated with the capture antibody by overnight incubation with 100 μL well^−1^ of mouse monoclonal anti-PLTX 73D3 antibody (20 μg mL^−1^) at 4 °C. Then, the wells were blocked with 200 μL of 2% skimmed milk (*w*/*v*) dissolved in PBS containing 0.1% Tween 20 (PBS-Tw) for 1 h at room temperature (RT) and incubated for 2 h at RT with 100 μL of PLTX solution (at different PLTX concentrations to obtain a calibration curve) or the microalgal extract solution in PBS-Tw (1:10). The wells were washed and incubated with the secondary antibody (100 μL well^−1^ of purified rabbit polyclonal anti-PLTX antibodies, 0.17 μg mL^−1^) for 2 h at RT. After washings, each well was incubated with the detection antibody (100 μL of horseradish peroxidase-conjugated goat anti-rabbit polyclonal antibodies, 1:2000) for 1 h at RT. After washings, the substrate and chromogen solution (3,3’,5,5’-tetramethylbenzidine, 60 μL) was added to each well and the colorimetric reaction was stopped after 30 min by 30 μL of 1 M H_2_SO_4_. The absorbance of each well solution was measured at 450 nm (Spectra photometer; Tecan Italia; Milan, Italy). PLTX equivalents in the microalgal extract were determined by translating the absorbance into concentration by extrapolation from a PLTX calibration curve and are reported as mean of three independent experiments performed in triplicate.

#### 5.4.3. Liquid Chromatography–High-Resolution Multiple Stage Mass Spectrometry (LC-HRMS^n^)

A hybrid linear ion trap LTQ Orbitrap XL^TM^ Fourier transform MS (FTMS) with an ESI ION MAX^TM^ source (Thermo-Fisher, San Josè, CA, USA) system coupled to a Dionex Ultimate 3000 quaternary system was used for analyzing the crude algal extract (injection volume = 5 µL). A Poroshell 120 EC-C18 (2.7 μm, 2.1 × 100 mm) (Agilent, USA) column kept at room temperature was used eluted with mobile phases (A = water and B = 95% acetonitrile/water), both added of 30 mM acetic acid. Flow was set at 0.2 mL min^-1^. A good chromatographic separation among most PLTX congeners was obtained by using a slow gradient elution: 28–29% B over 5 min, 29–30% B over 10 min, 30–100% B in 1 min, and held for 5 min [23].

Positive ion HR full scan MS experiments were acquired in the range *m*/*z* 800–1400 at a resolving power of 60,000 (FWHM at *m*/*z* 400). Ionization source parameters were the followings: Spray voltage = 4.8 kV, capillary temperature = 290 °C, capillary voltage = 17 V, sheath gas = 32 and auxiliary gas = 4 (arbitrary units), and tube lens voltage = 145 V. HR collision-induced dissociation (CID) MS^2^ experiments were acquired at a resolving power of 60,000 using a collision energy of 35%, isolation width of 4.0 Da, activation Q of 0.250, and activation time of 30 ms. The most intense peaks of the [M + H + Ca]^3+^ ion cluster of isobaric palytoxin and individual ovatoxins were used as precursors. The monoisotopic peak of each ion cluster was used for calculating elemental composition (Xcalibur software v2.0.7 at a mass tolerance constraint of 5 ppm). The isotopic pattern of each ion cluster was considered in ion assignment.

Extracted ion chromatograms of the [M + H + Ca]^3+^ ion of each known PLTX congener were used for quantitation. A not certified PLTX standard was used to prepare a calibration curve at five levels of concentration (100, 50, 25, 12.5, and 6.25 ng mL^−1^), which was used for OVTX and isobaric PLTX determination in the crude extract by assuming that their molar responses were similar to that of PLTX. Calibration curve equation was y = 31657x − 211166 and its linearity was expressed by *R*^2^ = 0.9987.

## Figures and Tables

**Table 1:** Overview of temporal and spatial distribution of *Ostreopsis* species with recorded abundances in the water column (cells L^−1^) and on macrophyte (cells g^−1^) in the Mediterranean Sea obtained from literature.

Year Month	Location	Authors	Species	Water Column (Cells L^−1^)	Macrophyte (Cells g^−1^)
1972	Villefranche-sur-Mer	Taylor’s personal communication cited in [32]	*O.* cf. *ovata*		
1979	Lebanese water	[33]	*Ostreopsis* sp.		
1984	Kaštela Bay	[30]	*O.* cf. *ovata*		
1994 Aug	Tyrrhenian Sea	[34]	*O.* cf. *ovata*	8000	
1995–1999	Catalan Sea (Garraf and Blanes harbor)	[35,36]	*Ostreopsis* spp.	78,000; 98,000	590,000
1997 July	Catalan Sea	[1]	*Ostreopsis* sp.		596,000
1998 Aug	Catalan Sea (Llavaneres)	[36]	*O.* cf. *ovata* *O. siamensis*	200,000	
1998 Aug	Coast of Tuscany	[37]	*O.* cf. *ovata*	50,000 (3,000,000,000 in the resuspended mat)	
2000 Oct	Gulf of Gabés, Tunisia	[38]	*O. siamensis*	≈150	
2001	Balearic islands	[36]	*Ostreopsis* spp.	25,000	
2001 July	Lebanese waters	[39]	*O. siamensis*	10,560	
2001 Aug–Sept	Gulf of Tunis, Tunisia	[40]	*O. siamensis*		3600
2002 Aug	Tyrrhenian Sea Marina di Massa	[41]	*O.*cf. *ovata*	10,550	
2003 Aug 2004 Sept	South Italy, coasts of Bari	[42]	*Ostreopsis* spp.	>1,000,000	
2003–2004 Sept	North Aegean Sea	[43]	*O. ovata*, *O.* cf. *siamensis*	16,000	405,000
2004 Aug	Catalan Sea	[36]	*O.* cf. *siamensis*, *O. ovata*	106,655	
2004 Aug	Balearic islands	[36]	*O.* cf. *siamensis, O. ovata*	1280	
2004 July	Tyrrhenian Sea, Gulf of Naples	[32]	*O. ovata*		
2004 Sept 2005 Sept	North Aegean Sea	[44]	*Ostreopsis* spp.	1000; 3600	39,493; 33,212
2005 July	Ligurian Sea, Gulf of Genoa	[22]	*O. ovata*	1,800,000	
2005 July	Alexandria, Egypt	[45]	*Ostreopsis* spp.		9053
2005 May	Aegean Sea (Gulf of Kalloni)	[46]	*O.* cf. *ovata*	600	
2005–2007	Egyptian Mediterranean waters	[45]	*O.* cf. *ovata*		
2006 Aug	Morgiret, Iles de Frioul, off the coast of Marseille, France	[47]	*Ostreopsis* spp.	900,000	
2006 July	French coast: Frioul Island, Marseille	[9]	*Ostreopsis* spp.	>30,000	
2006 July	Ligurian Sea	[24,48]	*O.* cf. *ovata*	87,000 (± 27,000)	2,541,000 (±588,000)
2006 July	Catalan Sea (monitoring of 14 beaches) (beach Ses Illeters	[49]	*Ostreopsis* spp. *(O.*cf. *ovata*, *O.* cf. *siamensis)*	34,445	
2006 July	Alexandria, Egypt	[45]	*Ostreopsis* spp.		≈3500
2006 July	Tunis Lake Bizerte	[50]	*O.* cf. *siamensis*	24,700	
2006 Sept	Ligurian Sea Gulf La Spezia	[51]	*O.* cf. *ovata*	12,000,000	
2006 Sept	Adriatic Sea Conero riviera	[52]	*O.* cf. *ovata*	2000	20,000
2006	Adriatic Sea Gulf of Trieste and close to Rovinj (Croatia)	[53]	*O.* cf. *ovata*		
2007 Aug, July; 2008 July, Oct; 2009 July, Aug	Balearic Sea	[2]	*O.* cf. *ovata*, *O.* cf. *siamensis*	33,908; 80,272; 385,601	2,600,239; 7,248,635; 4,008,204
2007–2008	Monaco (Larvotto beach)	[54]	*O.* cf. *ovata*	213,000	2,800,000
2007 Aug–Sept	NW Adriatic Sea (Conero Riviera)	[3]	*Ostreopsis* spp. *(O. ovata* and *O.* cf. *siamensis)*	25,000 (± 4000) (average values)	160,000 (±28,000) (average values)
2007 Aug, July; 2008 Aug; 2009 Sept, July	Gulf of Lion	[2]	*O.* cf. *ovata*	46,600; 36,900; 116,200	105,923; 186,480; 392,756
	Eastern Harbour of Alexandria, Egipt	[55]	*Ostreopsis* spp.		
2007 July	Alexandria, Egypt	[45]	*Ostreopsis* spp.		≈4500
2007 July	Tunis Lake Bizerte	[50]	*O.* cf. *siamensis*	37,500	
2007 July; 2008 July, Aug; 2009 July	Ligurian Sea	[2]	*O.* cf. *ovata*	43,278; 104,000; 16,100	1,592,511; 1,433,470; 1,610,462
2007 July–Aug	Morgiret, Iles de Frioul, off the coast of Marseille, France	[47]	*Ostreopsis* spp.	≈8000	≈100,000
2007 June–Aug	French coast: Aygulf Beach, Fréjus; Larvotto Beach, Monaco; Méjan Beach, Toulon	[9]	*Ostreopsis* spp.	>30,000	
2007 May–Aug	Catalan Sea	[3]	*Ostreopsis* spp. (*O. ovata*, *O.* cf. *siamensis)*	20,000 (±3000)	3,000,000 (±540,000)
2007 Oct	Adriatic Sea Conero Riviera	[4]	*O.*cf. *ovata*	25,200 (13,500,000 in the resuspended mat)	1,700,000
2007 Sept	South Adriatic (Puglia region)	[56]	*O.* cf. *ovata*	4900 (bottom water 421,200)	
2007 Sept–Oct 2009 Sept–Oct	Adriatic Sea (Ancona)	[2]	*O.* cf. *ovata*	25,279; 92,483	1,701,614; 1,626,621
2007–2010	NW Mediterranean Sea (Catalan coast)	[49]	*Ostreopsis* sp.		
2007–2010	Italian region Marche	ISPRA 2010, 2011 cited in [28]	*O.* cf. *ovata*	641,000–7,000,000	
2007–2011	Italian region Puglia	ISPRA 2010, 2011, 2012, cited in [28]	*O.* cf. *ovata*	36,400–7,500,000	
2008 Aug	Coast of Tuscany	[57]	*O.* cf. *ovata*	95,200	
2008 Aug	Ionian Sea (Puglia region)	[56]	*O.* cf. *ovata*	7680 (bottom water 160,000)	
2008 Aug	Abruzzo coast (Ortona)	[58]	*O.* cf. *ovata*	3600	
2008 Aug	Western Algiers area Bou-Ismaïl Bay waters	[59]	*Ostreopsis* spp.	3000	
2008 Aug–Sept	South Adriatic (Puglia region)	[56]	*O.* cf. *ovata*	304,000 (bottom water 5,000,000)	
2008 July	Catalan Sea (monitoring of 14 beaches) (beach Llavaneres)	[49,60]	*Ostreopsis* spp. *(O.* cf. *ovata*, *O.* cf. *siamensis)*	205,632	several millions (EBITOX)
2008 July–Sept	French coast: Marinière Beach, Villefranche; Réserve Beach, Nice; Frioul Island, Marseille	[9]	*Ostreopsis* spp.	>30,000	
2008 July 2009 Jan	Eastern Tunisia Mahdia	[61]	*O.* cf. *siamensis*		1–5 (average values)
2008 July–Aug	Morgiret, Iles de Frioul, off the coast of Marseille, France	[47]	*Ostreopsis* spp.	≈5,000,000	≈300,000
2008 June–Aug	Ligurian Sea Genoa; Villefranche-sur-Mer; Nice; Saint Raphael; Ramatuelle	[62]	*O.* cf. *ovata*	68,000; 7000; 12,000; 400; 3000	2,810,000; 8,540,000; 1,980,000; 20,000; 10,000
2008 June–Aug	Gulf of Lyon	[62]	*O.* cf. *ovata*	1000	60,000
2008–2009	Albania Butrinti lagoon	[63]	*Ostreopsis* spp.		
2009 Aug	Ionian Sea	[64]	*O.*cf. *ovata*	757,800 (±114,300) (average values)	422,300 (±120,000) (average values)
2009 July	SW Mediterranean Algerian beaches	[65]	*Ostreopsis* spp.	5920	20,000
2009 July	Catalan Sea (monitoring of 14 beaches) (beach Alguer)	[49,60]	*Ostreopsis* spp. *(O.*cf. *ovata*, *O.* cf. *siamensis)*	2400	
2009 July–Sept	French coast: Marinière Beach, Villefranche; Frioul Island, Marseille	[9]	*Ostreopsis* spp.	>30,000	
2009 July–Sept	Morgiret, Iles de Frioul, off the coast of Marseille, France	[47]	*Ostreopsis* spp.	≈120,000	≈400,000
2009 Oct, Sept	Adriatic Sea (North Eastern part	[2]	*O.* cf. *ovata*	280	333,793
2009 Sept	Adriatic Sea (Gulf of Trieste)	[66,67]	*O.* cf. *ovata*	3,076,416 6,700,000	
2009 Sept	Adriatic Sea (Conero Riviera)	[68]	*O.* cf. *ovata*	92,000	1,313,000
2009 Sept	Adriatic Sea (Conero Riviera)	[5,69]	*O.*cf. *ovata*	>120,000	>70,000
2010 Aug	Adriatic Sea (Conero Riviera)	[70]	*O.*cf. *ovata*	10,200	1,200,000
2010	Italian region Liguria	ISPRA 2011 cited in [28]	*O.* cf. *ovata*	10,200,000	
2010 Aug	Catalan Sea (monitoring of 14 beaches) (beach Castelldefels)	[49,60]	*Ostreopsis* spp. *(O.*cf. *ovata*, *O.* cf. *siamensis)*	1680	
2010 July–Aug	SW Mediterranean Algerian beaches	[65]	*Ostreopsis* spp.	21,680	79,000
2010 July–Aug	Genoa, Italy Quarto dei Mille	[71]	*O.* cf. *ovata*	20,670	733,678
2010 May–Dec	Lebanese waters	[39]	*O. siamensis*	about 250	
2010 Oct	Cesme Bay (Eastern Aegean coast)	[72]	*O.* cf. *ovata*	65,000	
2010 Sep–Oct	Adriatic Sea (northern Adriatic, public beach close to the city of Rovinj, Croatia	[31]	*O.* cf. *ovata*	42,600	334,306
2011 July	Villefranche-sur-Mer	[73]	*O.* cf. *ovata*	28,000	3,700,000
2011 July	Villefranche-sur-Mer	[73]	*O.* cf. *ovata*	70,000	490,000
2011 July	Genoa, Italy Quarto dei Mille	[71]	*O.* cf. *ovata*	4770	412,930
1997–2012 Oct–Nov	Tunisia (Gulf of Gabes)	[74]	*O.* cf. *siamensis*	5000–8000	
2012 July	Genoa, Italy Quarto dei Mille	[71]	*O.* cf. *ovata*	24,740	1,919,740
2012 July–Aug	Sardinian coast, Italy	[75]	*O.* cf. *ovata*	1100	
2013 July–Aug	Genoa, Italy Quarto dei Mille	[71]	*O.* cf. *ovata*	24,520	973,882
2016 Aug	Catalan coast Sant Andreu de Llavaneres	[76]	*O.* cf. *ovata*	≈500,000	≈500,000
2014 July	Genoa, Italy Quarto dei Mille	[71]	*O.* cf. *ovata*	7340	218,365
2014	Greece and Cyprus coasts	[77]	New genotype *Ostreopsis* sp.		
2014	Southern Mediterranean, Bizerte Bay	[78]	*O.* cf. *ovata*		
2015 July	Genoa, Italy Quarto dei Mille	[71]	*O.* cf. *ovata*	51,719	2,289,100
2015 June–July	Cyprus and Lebanon	[8]	*O. fattorussoi*	840	28,000
2016	Italian region Veneto	[79]	*O.* cf. *ovata*	820	
2016 Aug	Italian region Puglia	[79]	*O.* cf. *ovata*	7,362,000	
2016 Aug	Italian region Calabria	[79]	*O.* cf. *ovata*	4000	6,878
2016 Aug	Sardinia	[79]	*O.* cf. *ovata*	40,333	841,270
2016 July	Italian region Campania	[79]	*O.* cf. *ovata*	39,362	371,696
2016 July	Italian region Lazio	[79]	*O.* cf. *ovata*	141,140	10,008,076
2016 July	Italian region Tuscany	[79]	*O.* cf. *ovata*	634,800	
2016 July	Sicily	[79]	*O.* cf. *ovata*	225,503 ± 20,976	410,580 ± 54,010
2016 July–Aug	Italian region Liguria	[79]	*O.* cf. *ovata*	101,760	349,463
2016 Sept	Italian region Marche	[79]	*O.* cf. *ovata*	6,860,000	58,960

**Table 2:** Morphological characteristics of *O.* cf. *ovata* cells. Average values (Av) ± standard deviation (SD) of dorsoventral diameter (DV), anteroposterior diameter (AP), and ratio DV/AP, with minimum (min) and maximum (max) (*n* = 58).

Basic Statistic	DV (µm)	AP (µm)	DV/AP
Av ± SD	54.81 ± 5.07	25.41 ± 2.27	2.17 ± 0.20
min–max	40.00–63.73	21.20–31.80	1.57–2.54

**Table 3:** Phytoplankton abundance and community composition in the water column during *Ostreopsis* bloom in Kaštela Bay in 2015. The table provides the abundances recorded in replicates with maximal abundances of *O*. cf. *ovata*.

Phytoplankton Species	Abundance (Cells L^−1^)	
	18 September	1 October
**Diatoms**		
*Bacteriastrum* sp.	2560	5120
*Chaetoceros affinis*		
*Chaetoceros* sp.	40,960	
*Cylindrotheca closterium*	7680	23,040
*Dactyliosolen fragilissimus*		17,920
*Guinardia delicatula*	19,200	
*Guinardia flaccida*	1280	1280
*Guinardia striata*	20,480	76,800
*Hemiaulus haucki*		2560
*Leptocylindrus danicus*	14,080	12,800
*Leptocylindrus mediterraneus*	1280	
*Licmophora flabelata*	1280	1280
*Navicula* sp.	17,920	10,240
Pennatae indeterm	10,240	12,800
*Pleurosigma* sp.	1280	1280
*Proboscia alata*	10,240	7680
*Pseudo-nitzschia* spp.	98,560	136,960
*Striatella unipunctata*		1280
*Thalassionema nitzschioides*	6400	5120
**Dinoflagellates**		
*Alexandrium minutum*		1280
*Amphidinium carterae*		1280
*Coolia* sp.	1120	1600
*Dinophysis fortii*		1280
*Gymnodinium* sp.1		2560
*Gymnodinium* sp.2 (<20 µm)	5120	
*Gyrodinium fusiforme*		1280
*Ostreopsis* sp.	28,560	1920
*Prorocentrum* sp.		1280
**Coccolithophorids**		
*Rhabdosphaera clavigera*	1280	
*Syracosphaera pulchra*		1280
**Euglenophyta**		
*Eutreptiella* sp.	1280	

**Table 4:** Individual and total toxin concentration measured in *O.* cf. *ovata* cells by LC-HRMS (pg cell^−1^) and total toxin content measured by the indirect sandwich ELISA (pg PLTX eq cell^−1^).

LC-HRMS (pg cell^−1^)						ELISA (pg PLTX eq. cell^−1^)
OVTX-a	OVTX-b	OVTX-c	OVTX-d/e	Isobaric PLTX	Total	
3.6	1.3	0.2	1.1	0.1	6.3	5.6

## References

[1] Vila M., Garcés E., Masó M. (2001). Potentially toxic epiphytic dinoflagellates assemblages on macroalgae in the NW Mediterranean. Aquat. Microb. Ecol..

[2] Mangialajo L., Ganzin N., Accoroni S., Asnaghi V., Blanfuné A., Cabrini M., Cattaneo-Vietti R., Chavanon F., Chiantore M., Cohu S. (2011). Trends in *Ostreopsis* proliferation along the Northern Mediterranean coasts. Toxicon.

[3] Battocchi C., Totti C., Vila M., Masó M., Capellacci S., Accoroni S., Reñé A., Scardi M., Penna A. (2010). Monitoring toxic microalgae *Ostreopsis* (dinoflagellate) species in coastal waters of the Mediterranean Sea using molecular PCR-based assay combined with light microscopy. Mar. Pollut. Bull..

[4] Totti C., Accoroni S., Cerino F., Cucchiari E., Romagnoli T. (2010). *Ostreopsis ovata* bloom along the Conero Riviera (northern Adriatic Sea): Relationships with environmental conditions and substrata. Harmful Algae.

[5] Penna A., Fraga S., Battocchi C., Casabianca S., Perini F., Cappellacci S., Casabianca A., Riobó P., Giacobbe M.G., Totti C. (2012). Genetic diversity of the genus *Ostreopsis* Schmidt: Phylogeographical considerations and molecular methodology applications for field detection in the Mediterranean Sea. Cryptogamie Algol..

[6] Penna A., Vila M., Fraga S., Giacobbe M.G., Andreoni F., Riobó P., Vernesi C. (2005). Characterization of *Ostreopsis* and *Coolia* (Dinophyceae) isolates in the Western Mediterranean Sea based on morphology, toxicity and internal transcribed spacer 5.8 S rDNA sequences. J. Phycol..

[7] Penna A., Fraga S., Battocchi C., Casabianca S., Giacobbe M.G., Riobó P., Vernesi C. (2010). A phylogeographical study of the toxic benthic dinoflagellate genus *Ostreopsis* Schmidt. J. Biogeogr..

[8] Accoroni S., Romagnoli T., Penna A., Capellacci S., Ciminiello P., Dell’Aversano C., Tartaglione L., Abboud-Abi Saab M., Giussani V., Asnaghi V. (2016). *Ostreopsis fattorussoi* sp. nov. (Dinophyceae), a new benthic toxic *Ostreopsis* species from the eastern Mediterranean Sea. J. Phycol..

[9] Tichadou L., Glaizal M., Armengaud A., Grossel H., Lemée R., Kantin R., Lasalle J.L., Drouet G., Rambaud L., Malfait P. (2010). Health impact of unicellular algae of the *Ostreopsis* genus blooms in the Mediterranean Sea: Experience of the French Mediterranean coast surveillance network from 2006 to 2009. Clin. Toxicol..

[10] Durando P., Ansaldi F., Oreste P., Moscatelli P., Marensi L., Grillo C., Gasparini R., Icardi G. (2007). *Ostreopsis ovata* and human health: Epidemiological and clinical features of respiratory syndrome outbreaks from a two year syndromic surveillance, 2005–2006, in northwest Italy. Euro Surveill..

[11] Del Favero G., Sosa S., Pelin M., D’Orlando E., Florio C., Lorenzon P., Poli M., Tubaro A. (2012). Sanitary problems related to the presence of *Ostreopsis* spp. in the Mediterranean Sea: A multidisciplinary scientific approach. Ann. Ist. Super. Sanita.

[12] Ciminiello P., Dell’Aversano C., Dello Iacovo E., Fattorusso E., Forino M., Tartaglione L., Benedettini G., Onorari M., Serena F., Battocchi C., Casabianca S., Penna A. (2014). First finding of *Ostreopsis* cf. *ovata* toxins in marine aerosols. Environ. Sci. Technol..

[13] Tubaro A., Durando P., Del Favero G., Ansaldi F., Icardi G., Deeds J.R., Sosa S. (2011). Case definitions for human poisonings postulated to palytoxins exposure. Toxicon.

[14] Casabianca S., Casabianca A., Riobó P., Franco J., Vila M., Penna A. (2013). Quantification of the toxic dinoflagellate *Ostreopsis* spp. by qPCR assay in marine aerosol. Environ. Sci. Technol..

[15] Fukui M., Murata M., Inoue A., Gawel M., Yasumoto T. (1987). Occurrence of palytoxin in the trigger fish *Melichtys vidua*. Toxicon.

[16] Kodama A.M., Hokama Y., Yasumoto T., Fukui M., Manea S.J., Sutherland N. (1989). Clinical and laboratory findings implicating palytoxin as cause of ciguatera poisoning due to *Decapterus macrosoma* (mackerel). Toxicon.

[17] Onuma Y., Satake M., Ukena T., Roux J., Chanteau S., Rasolofonirina N., Ratsimaloto M., Naoki H., Yasumoto T. (1999). Identification of putative palytoxin as the cause of clupeotoxism. Toxicon.

[18] Taniyama S., Arakawa O., Terada M., Nishio S., Takatani T., Mahmud Y., Noguchi T. (2003). *Ostreopsis* sp., a possible origin of palytoxin (PTX) in parrotfish *Scarus ovifrons*. Toxicon.

[19] Wu M.L., Yang C.C., Deng J.F., Wang K.Y. (2014). Hyperkalemia, hyperphosphatemia, acute kidney injury, and fatal dysrhythmias after consumption of palytoxin-contaminated goldspot herring. Ann. Emerg. Med..

[20] Yasumoto T., Yasumura D., Ohizumi Y., Takahashi M., Alcala A.C., Alcala L.C. (1986). Palytoxin in two species of xanthid crab from the Philippines. Agric. Biol. Chem..

[21] Alcala A.C., Alcala L.C., Garth J.S., Yasumura D., Yasomoto T. (1988). Human fatality due to ingestion of the crab *Demania rynaudii* that contained a palytoxin-like toxin. Toxicon.

[22] Ciminiello P., Dell’Aversano C., Fattorusso E., Forino M., Magno G.S., Tartaglione L., Grillo C., Melchiorre N. (2006). The Genoa 2005 outbreak. Determination of Putative Palytoxin in Mediterranean *Ostreopsis ovata* by a New Liquid Chromatography Tandem Mass Spectrometry Method. Anal. Chem..

[23] García-Altares M., Tartaglione L., Dell’Aversano C., Carnicer O., de la Iglesia P., Forino M., Diogéne J., Ciminiello P. (2015). The Novel Ovatoxin-g and Isobaric Palytoxin (so far referred to as Putative Palytoxin) from *Ostreopsis* cf. *ovata* (NW Mediterranean Sea): Structural Insights by LC-High Resolution MSn. Anal. Bioanal. Chem..

[24] Ciminiello P., Dell’Aversano C., Fattorusso E., Forino M., Tartaglione L., Grillo C., Melchiorre N. (2008). Putative palytoxin and its new analogue, ovatoxin-a, in *Ostreopsis ovata* collected along the Ligurian coasts during the 2006 toxic outbreak. J. Am. Soc. Mass Spectrom..

[25] Ciminiello P., Dell’Aversano C., Dello Iacovo E., Fattorusso E., Forino M., Grauso L., Tartaglione L., Guerrini F., Pistocchi R. (2010). Complex palytoxin-like profile of *Ostreopsis ovata*. Identification of four new ovatoxins by high-resolution liquid chromatography/mass spectrometry. Rapid Commun. Mass Sp..

[26] Ciminiello P., Dell’Aversano C., Dello Iacovo E., Fattorusso E., Forino M., Grauso L., Tartaglione L., Guerrini F., Pezzolesi L., Pistocchi R. (2012). Isolation and structure elucidation of ovatoxin-a, the major toxin produced by *Ostreopsis ovata*. J. Am. Chem. Soc..

[27] Rossi R., Castellano V., Scalco E., Serpe L., Zingone A., Soprano V. (2010). New palytoxin-like molecules in Mediterranean *Ostreopsis* cf. *ovata* (dinoflagellates) and in Palythoa tuberculosa detected by liquid chromatography-electrospray ionization time-of-flight mass spectrometry. Toxicon.

[28] Funari E., Manganelli M., Testai E. (2015). *Ostreopsis* cf. *ovata* blooms in coastal water: Italian guidelines to assess and manage the risk associated to bathing waters and recreational activities. Harmful Algae.

[29] Pelin M., Forino M., Brovedani V., Tartaglione L., Dell’Aversano C., Pistocchi R., Poli M., Sosa S., Florio C., Ciminiello P. (2016). Ovatoxin-a, a palytoxin analogue isolated from *Ostreopsis* cf. *ovata* Fukuyo: Cytotoxic activity and ELISA detection. Environ. Sci. Technol..

[30] Marasović I. (1990). Proportion of dinoflagellates in the phytoplankton community of the Middle Adriatic with special regard to “red tide” and toxic species (in Croatian). Ph.D. Thesis.

[31] Pfannkuchen M., Godrijan J., Marić Pfannkuchen D., Iveša L., Kružić P., Ciminiello P., Dell’Aversano C., Dello Iacovo E., Fattorusso E., Forino M. (2012). Toxin-producing *Ostreopsis* cf. *ovata* are likely to bloom undetected along Coastal Areas. Environ. Sci. Technol..

[32] Zingone A., Siano R., Alelio D.D., Sarno D. (2006). Potentially toxic and harmful microalgae from coastal waters of the Campania region (Tyrrhenian Sea, Mediterranean Sea). Harmful Algae.

[33] Abboud-Abi Saab M. (1989). Les dinoflagellés des eaux côtières libanaises-espèces rares ou nouvelles du phytoplancton marin. Leban. Sci. Bull..

[34] Tognetto L., Bellato S., Moro I., Andreoli C. (1995). Occurrence of *Ostreopsis ovata* (Dinophyceae) in the Tyrrhenian Sea during summer 1994. Bot. Mar..

[35] Vila M., Camp J., Garcés E., Masó M., Delgado M. (2001). High resolution spatial-temporal detection of HABs in confined waters of the NW Mediterranean. J. Plankton Res..

[36] Vila M., Masó M., Sampedro N., Illoul H., Arin L., Garcés E., Giacobbe M.G., Alvarez J., Camp J. (2008). The genus *Ostreopsis* in the recreational waters of the Catalan Coast and Balearic Islands (NW Mediterranean Sea): Is this the origin of human respiratory difficulties?. Proceedings of the 12th International Conference of Harmful Algae.

[37] Sansoni G., Borghini B., Camici G., Casotti M., Righini P., Rustighi C. (2003). Fioriture algali di *Ostreopsis ovata* (Gonyaulacales: Dinophyceae): Un problema emergente. Biologia Ambientale.

[38] Turki S., Harzallah A., Sammari C. (2006). Occurrence of harmful dinoflagellates in two different Tunisian ecosystems: The lake of Bizerte and the Gulf of Gabes. Cah. Biol. Mar..

[39] Abboud-Abi Saab M., Fakhri M., Kassab M.T., Matar N. (2013). Seasonal and spatial variations of the dinoflagellate *Ostreopsis siamensis* in the Lebanese coastal waters (Eastern Mediterranean). Cryptogamie Algol..

[40] Turki S. (2005). Distribution of toxic dinoflagellates along the leaves of seagrass *Posidonia oceanica* and *Cymodocea nodosa* from the Gulf of Tunis. Cah. Biol. Mar..

[41] Simoni F., Gaddi A., Di Paolo C., Lepri L. (2003). Harmful epiphytic dinoflagellates on Tyrrhenian Sea. Harmful Algae News.

[42] Gallitelli M., Ungaro N., Addante L.M., Procacci V., Silver N.G., Sabbà C. (2005). Respiratory illness as a reaction to tropical algal blooms occurring in a temperate climate. Jama.

[43] Aligizaki K., Nikolaidis G. (2006). The presence of the potentially toxic genera *Ostreopsis* and *Coolia* (Dinophyceae) in the North Aegean Sea, Greece. Harmful Algae.

[44] Aligizaki K., Katikou P., Nikolaidis G., Panou A. (2008). First episode of shellfish contamination by palytoxin-like compounds from *Ostreopsis species* (Aegean Sea, Greece). Toxicon.

[45] Ismael A., Halim Y. (2012). Potentially harmful *Ostreopsis* spp. in the coastal waters of Alexandria- Egypt. Medit. Mar. Sci..

[46] Spatharis S., Dolapsakis N.P., Economou-Amilli A., Tsirtsis G., Danielidis D.B. (2009). Dynamics of potentially harmful microalgae in a confined Mediterranean Gulf - Assessing the risk of bloom formation. Harmful Algae.

[47] Amzil Z., Sibat M., Chomerat N., Grossel H., Marco-Miralles F., Lemee R., Nezan E., Sechet V. (2012). Ovatoxin-a and palytoxin accumulation in seafood in relation to *Ostreopsis* cf. *ovata* blooms on the French. Mar. Drugs.

[48] Mangialajo L., Bertolotto R., Cattaneo-Vietti R., Chaintore M., Grillo C., Lemee R., Melchiorre N., Moretto P., Povero P., Ruggieri N. (2008). The toxic benthic dinoflagellate *Ostreopsis ovata*: Quantification of proliferation along the coastline of Genoa, Italy. Mar. Pollut. Bull..

[49] Vila M., Arin L., Battocchi C., Bravo I., Fraga S., Penna A., Reñé A., Riobó P., Rodriguez F., Sala M.M. (2012). Management of *Ostreopsis* blooms in recreational waters along the Catalan coast (NW Mediterranean Sea): Cooperation between a research project and a monitoring program. Cryptogamie Algol..

[50] Turki S., Balti N., Aissaoui A., Armi Z. (2010). *Ostreopsis* cf. *siamensis* proliferations in coastal water of Bizerte, Northern Tunisia. Harmful Algae News.

[51] Abbate M., Bordone A., Cerrati G., Lisca A., Peirano A. (2007). Variabilità della distribuzione e densità di *Ostreopsis ovata* nel Golfo della Spezia. Biol. Mar. Mediterr..

[52] Totti C., Cucchiari E., Romagnoli T., Penna A. (2007). Bloom of *Ostreopsis ovata* on the Conero riviera (NW Adriatic Sea). Harmful Algae News.

[53] Monti M., Minocci M., Beran A., Iveša L. (2007). First record of *Ostreopsis* cfr. *ovata* on macroalgae in the Northern Adriatic Sea. Mar. Pollut. Bull..

[54] Cohu S., Thibaut T., Mangialajo L., Labat J.P., Passafiume O., Blanfuné A., Simon N., Cottalorda J.M., Lemée R. (2011). Occurrence of the toxic dinoflagellate *Ostreopsis* cf. *ovata* in relation with environmental factors in Monaco (NW Mediterranean). Mar. Pollut. Bull..

[55] Halim Y. (2007). First IOC/HANA workshop on harmful algal blooms in North Africa. Harmful Algae News.

[56] Ungano N., Assennato G., Blonda M., Cudillo B., Petruzzelli M.R., Mariani M., Pastorelli A.M., Aliquò M.R., D’Angela A., Aiello C. (2010). Occurrence of the potentially toxic dinoflagellate *Ostreopsis ovata* along the apulian coastal areas (Southern Italy) and relationship with anthropogenic pollution. Fresenius Environ. Bull..

[57] Milandri A., Ceredi A., Riccardi E., Gasperetti L., Susini F., Casotti M., Faiman L., Pigozzi S., Pagou K., Hallegraeff G. (2013). Impact of *Ostreopsis ovata* on marine benthic communities: Accumulation of palytoxins in suffering mussels, sea urchins and octopuses from Italy. Abstract Book of the 14th International Conference on Harmful Algae, Hersonissons-Crete, Greece, 1–5 November 2010.

[58] Ingarao C., Lanciani G., Teodori A., Pagliani T. (2009). First presence of *Ostreopsis* cf. *ovata* (Dinophyceae) along Abruzzo coasts (W Adriatic Sea). Biol. Mar. Medit..

[59] Illoul H., Masó M., Demestre M., Fortuño J.M., De Juan S. Harmful algae in Bou-Ismaïl Bay coastal waters during August 2008 cruise (Algerian coast). (AECI/ MESRS A/010153/07 Project). Proceedings of the IOC/HANA Second workshop on Harmful Algal Blooms in North Africa.

[60] Vila M., Riobó P., Bravo I., Masó M., Penna A., Reñé A., Sala M., Battocchi C., Fraga S., Rodriguez F., Pagou K., Hallegraeff G. (2013). A three-year time series of toxic *Ostreopsis* blooming in a NW Mediterranean coastal site: Preliminary results. Proceedings of the 14th International Conference on Harmful Algae.

[61] Mabrouk L., Hamza A., Brahim M.B., Bradai M.N. (2011). Temporal and depth distribution of microepiphytes on *Posidonia oceanica* (L.) Delile leaves in a meadow off Tunisia. Mar. Ecol..

[62] Cohu S., Mangialajo L., Thibaut T., Blanfuné A., Marro S., Lemée R. (2013). Proliferation of the toxic dinoflagellate *Ostreopsis* cf. *ovata* in relation to depth, biotic substrate and environmental factors in the North West Mediterranean Sea. Harmful Algae.

[63] Bushati M., Koni E., Miho A., Bregaj M. (2010). Temporal distribution of potentially toxic algae (dinoflagellates and diatoms) in Butrinti lagoon. Nat. Montenegr..

[64] Pagliara P., Caroppo C. (2012). Toxicity assessment of *Amphidinium carterae*, *Coolia* cfr. *monotis* and *Ostreopsis* cfr. *ovata* (Dinophyta) isolated from the northern Ionian Sea (Mediterranean Sea). Toxicon.

[65] Illoul H., Hernández F.R., Vila M., Adjas N., Younes A.A., Bournissa M., Koroghli A., Marouf N., Rabia S., Ameur F.L.K. (2012). The genus *Ostreopsis* along the Algerian coastal waters (SW Mediterranean Sea) associated with a human respiratory intoxication episode. Cryptogamie Algol..

[66] Blasutto O., Celio M., Honsell G., Suraci C., Venuti M., Zanolin B., Acquavita A., Mattassi G., Pagou K., Hallegraeff G. (2013). Gulf of Trieste, northern Adriatic Sea: First record of *Ostreopsis ovata* bloom. Proceedings of the 14th International Conference on Harmful Algae.

[67] Honsell G., De Bortoli M., Boscolo S., Dell’Aversano C., Battocchi C., Fontanive G., Penna A., Berti F., Sosa S., Yasumoto T. (2011). Harmful dinoflagellate *Ostreopsis* cf. *ovata* Fukuyo: Detection of ovatoxins in field samples and cell immunolocalization using antipalytoxin antibodies. Environ. Sci. Technol..

[68] Accoroni S., Romagnoli T., Colombo F., Pennesi C., Gioia C., Camillo D., Marini M., Battocchi C., Ciminiello P., Dell C. (2011). *Ostreopsis* cf. *ovata* bloom in the northern Adriatic Sea during summer 2009: Ecology, molecular characterization and toxin profile. Mar. Pollut. Bull..

[69] Perini F., Casabianca A., Battocchi C., Accoroni S., Totti C., Penna A. (2011). New approach using the real-time PCR method for estimation of the toxic marine dinoflagellate *Ostreopsis* cf. *ovata* in marine environment. PLoS ONE.

[70] Accoroni S., Colombo F., Pichierri S., Romagnoli T., Marini M., Battocchi C., Penna A., Totti C. (2012). Ecology of *Ostreopsis* cf. *ovata* blooms in the northwestern Adriatic Sea. Cryptogamie Algol..

[71] Giussani V., Regionale L.A., Asnaghi V. (2017). Management of harmful benthic dinoflagellates requires targeted sampling methods and alarm thresholds. Harmful Algae.

[72] Bizsel N., Aligizaki K., Chiantore M., Lemée R., Mangialajo L. (2011). Detection of *Ostreopsis* cf. *ovata* in coastal waters of Turkey (East Aegean Sea). Proceedings of the International Conference on Ostreopsis Development.

[73] Brissard C., Herrenknecht C., Séchet V., Hervé F., Pisapia F., Harcouet J., Lémée R., Chomérat N., Hess P., Amzil Z. (2014). Complex toxin profile of French Mediterranean *Ostreopsis* cf. *ovata* strains, seafood accumulation and ovatoxins prepurification. Mar. Drugs.

[74] Abdennadher M., Zouari A.B., Sahnoun W.F., Alverca E., Penna A., Hamza A. (2017). *Ostreopsis* cf. *ovata* in the Gulf of Gabès (south-eastern Mediterranean Sea): Morphological, molecular and ecological characterization. Harmful Algae.

[75] Satta C.T., Padedda B.M., Stacca D., Simeone S., De Falco G., Penna A., Capellacci S., Pulina S., Perilli A., Sechi N. (2014). Assessment of harmful algal species using different approaches: The case study of the Sardinian coasts. AIOL.

[76] Vila M., Abós-Herràndiz R., Isern-Fontanet J., Àlvarez J., Berdalet E. (2016). Establishing the link between *Ostreopsis* cf. *ovata* blooms and human health impacts using ecology and epidemiology. Sci. Mar..

[77] Giussani V., Kletou D., Casabianca S., Cappellacci S., Asnaghi V., Penna A., Ciminiello P., Dell’Aversano C., Mazzeo A., Tartaglione L. (2014). New *Ostreopsis* species recorded along Cyprus coasts: Toxic effect and preliminary characterization of chemical-molecular aspects. Abstract book of 16th International Conference on Harmful Algae, Wellington, New Zealand, 27–31 October 2014.

[78] Ben-Gharbia H., Yahia O.K.D., Amzil Z., Chomérat N., Abadie E., Masseret E., Sibat M., Triki H.Z., Nouri H., Laabir M. (2016). Toxicity and growth assessments of three thermophilic benthic dinoflagellates (*Ostreopsis* cf. *ovata*, *Prorocentrum lima* and *Coolia monotis*) developing in the Southern Mediterranean basin. Toxins.

[79] (2017). ISPRA 2017, Monitoraggio della microalga potenzialmente tossica *Ostreopsis* cf. *ovata* lungo le coste italiane. Anno 2016.

[80] Penna A., Battocchi C., Capellacci S., Fraga S., Aligizaki K., Lemée R., Vernesi C. (2014). Mitochondrial, but not rDNA, genes fail to discriminate dinoflagellate species in the genus *Ostreopsis*. Harmful Algae.

[81] Accoroni S., Totti C. (2016). The toxic benthic dinoflagellates of the genus *Ostreopsis* in temperate areas: A review. Adv. Oceanogr. Limnol..

[82] Balech E. (1956). E’tude des Dinoflagelle´s du sable de Roscoff. Rev. Algol..

[83] Fukuyo Y. (1981). Taxonomical study on benthic dinoflagellates collected in coral reefs. Bull. Japan. Soc. Sci. Fish..

[84] Besada E.G., Loeblich L.A., Loeblich A.R. (1982). Observations on tropical, benthic dinoflagellates from ciguatera-endemic areas: *Coolia*, *Gambierdiscus*, and *Ostreopsis*. Bull. Mar. Sci..

[85] Fraga S., Rodríguez F., Caillaud A., Diogène J., Raho N., Zapata M. (2011). *Gambierdiscus excentricus* sp. nov. (Dinophyceae), a benthic toxic dinoflagellate from the Canary Islands (NE Atlantic Ocean). Harmful Algae.

[86] Escalera L., Benvenuto G., Scalco E., Zingone A., Montresor M. (2014). Ultrastructural Features of the Benthic Dinoflagellate *Ostreopsis* cf. *ovata* (Dinophyceae). Protist.

[87] Faust M.A., Morton S.L., Quod J.P. (1996). Further study of marine dinoflagellates: The genus *Ostreopsis* (Dinophyceae). J. Phycol..

[88] Sato S., Nishimura T., Uehara K., Sakanari H., Tawong W., Hariganeya N., Smith K., Rhodes L., Yasumoto T., Taira Y. (2011). Phylogeography of *Ostreopsis* along West Pacific coast, with special reference to a novel clade from Japan. PLoS ONE.

[89] Kang N.S., Jeong H.J., Lee S.Y., Lim A.S., Lee M.J., Kim H.S., Yih W. (2013). Morphology and molecular characterization of the epiphytic benthic dinoflagellate *Ostreopsis* cf. *ovata* in the temperate waters off Jeju Island, Korea. Harmful Algae.

[90] Simoni F., Di Paolo C., Gori L., Lepri L., Mancino A., Falaschi A. (2004). Further investigation on blooms of *Ostreopsis ovata*, *Coolia monotis*, *Prorocentrum lima* on the macroalgae of artificial and natural reefs in the Northern Tyrrhenian Sea. Harmful Algae News.

[91] Congestri R., Penna A., Zingone A. (2006). BENTOX-NET: A research and management initiative on *Ostreopsis* spp. and other benthic microalgal blooms along the Italian coast. Harmful Algae News.

[92] Hariganeya N., Tanimoto Y., Yamaguchi H., Nishimura T., Tawong W., Sakanari H., Yoshimatsu T., Sato S., Preston C.M., Adachi M. (2013). Quantitative PCR Method for Enumeration of Cells of Cryptic Species of the Toxic Marine Dinoflagellate *Ostreopsis* spp. in Coastal Waters of Japan. PLoS ONE.

[93] Ramos V., Salvi D., Machado J.P., Vale M., Azevedo J., Vasconcelos V. (2015). Culture-Independent Study of the Late-Stage of a Bloom of the Toxic Dinoflagellate *Ostreopsis* cf. *ovata*: Preliminary Findings Suggest Genetic Differences at the Sub-Species Level and Allow ITS2 Structure Characterization. Toxins.

[94] Casabianca S., Perini F., Casabianca A., Battocchi C., Giussani V., Chiantore M., Penna A. (2014). Monitoring toxic *Ostreopsis* cf. *ovata* in recreational waters using a qPCR based assay. Mar. Pollut. Bull..

[95] Guerrini F., Pezzolesi L., Feller A., Riccardi M., Ciminiello P., Dell’Aversano C., Tartaglione L., Dello Iacovo E., Fattorusso E., Forino M. (2010). Comparative growth and toxin profile of cultured *Ostreopsis ovata* from the Tyrrhenian and Adriatic Seas. Toxicon.

[96] Grbec B., Morović M., Kušpilić G., Matijević S., Matić F., Beg Paklar G., Ninčević Ž. (2009). The relationship between the atmospheric variability and productivity in the Adriatic Sea area. J. Mar. Biol. Assoc. UK.

[97] Occhipinti-Ambrogi A. (2007). Global change and marine communities: Alien species and climate change. Mar. Pollut. Bull..

[98] Granéli E., Vidyarathna N.K., Funari E., Cumaranatunga P.R.T., Scenati R. (2011). Can increases in temperature stimulate blooms of the toxic benthic dinoflagellate *Ostreopsis ovata*?. Harmful Algae.

[99] Grbec B., Matić F., Beg Paklar G., Morović M., Popović R., Vilibić I. (2018). Long-Term Trends, Variability and Extremes of In Situ Sea Surface Temperature Measured Along the Eastern Adriatic Coast and its Relationship to Hemispheric Processes. Pure Appl. Geophys..

[100] Herring S.C., Hoell A., Hoerling M.P., Kossin J.P., Schreck C.J., Stott P.A. (2016). Explaining Extreme Events of 2015 from a Climate Perspective. Bull. Am. Meteorol. Soc..

[101] Meroni L., Chiantore M., Petrillo M., Asnaghi V. (2018). Habitat effects on *Ostreopsis* cf. *ovata* bloom dynamics. Harmful Algae.

[102] Tartaglione L., Dello Iacovo E., Mazzeo A., Casabianca S., Ciminiello P., Penna A., Dell’Aversano C. (2017). Variability in Toxin Profiles of the Mediterranean *Ostreopsis* cf. *ovata* and in Structural Features of the Produced Ovatoxins. Environ. Sci. Technol..

[103] Utermöhl H. (1958). Zur Vervollkommnung der quantitativen Phytoplankton Methodik. Mit. Int. Ver. Theor. Angew. Limnol..

[104] Vassalli M., Penna A., Sbrana F., Casabianca S., Gjeci N., Capellacci S., Asnaghi V., Ottaviani E., Giussani V., Pugliese L. (2018). Intercalibration of counting methods for *Ostreopsis* spp. blooms in the Mediterranean Sea. Ecol. Indic..

[105] Boscolo S., Pelin M., De Bortoli M., Fontanive G., Barreras A., Berti F., Sosa S., Chaloin O., Bianco A., Yasumoto T. (2013). Sandwich ELISA assay for the quantitation of palytoxin and its analogs in natural samples. Environ. Sci. Technol..

